# LncRNA TINCR rs2288947 Polymorphism as a Genetic Susceptibility Factor for Diabetic Retinopathy

**DOI:** 10.1155/joph/2631055

**Published:** 2026-05-14

**Authors:** Jinling Liu, Jing Song, Shasha Han, Yuanyuan Li, Ping Chen, Qian Liu, Na Liu, Min Wu

**Affiliations:** ^1^ Department of Ocular Trauma, Hebei Medical University Third Hospital, Shijiazhuang, 050051, Hebei, China; ^2^ Department of Ophthalmology, Characteristic Medical Center of Chinese People’s Armed Police Force, Tianjin, 300161, China; ^3^ Department of Ophthalmology, Dongying People’s Hospital, Dongying, 257091, Shandong, China, dysrmyy.com/; ^4^ Department of Health Care, Dongying People’s Hospital, Dongying, 257091, Shandong, China, dysrmyy.com/; ^5^ Department of Ophthalmology, Hekou District People’s Hospital, Dongying, 257200, Shandong, China; ^6^ Department of Ophthalmology, Daping Hospital, Army Medical University, Chongqing, 400042, China, tmmu.edu.cn

**Keywords:** biomarker, diabetic retinopathy, genetic susceptibility, lncRNA TINCR, single-nucleotide polymorphism

## Abstract

**Background:**

Long noncoding RNAs (lncRNAs) are critically involved in the pathogenesis of diabetic retinopathy (DR). Nevertheless, the role of lncRNA TINCR in DR has not been fully elucidated.

**Objective:**

This study aimed to examine the relationship between the lncRNA TINCR polymorphism rs2288947 and DR susceptibility in a Chinese Han population with Type 2 diabetes mellitus (T2DM) and to explore the diagnostic potential of plasma lncRNA TINCR levels.

**Methods:**

A case‐control study was conducted with 507 participants (255 NPDR patients and 252 T2DM controls). Plasma TINCR expression was measured via qRT‐PCR, and rs2288947 genotyping was performed using the TaqMan assay. Multivariate logistic regression and ROC analyses were applied to evaluate diagnostic value and risk associations.

**Results:**

LncRNA TINCR expression was significantly elevated and served as an independent risk factor in NPDR patients and exhibited high diagnostic value. The *G* allele of rs2288947 was associated with increased NPDR risk and higher lncRNA TINCR expression. Carriers of GA/GG genotypes showed upregulated lncRNA TINCR, which correlated with longer diabetes duration, elevated HbA1c, reduced HDL‐C, and impaired renal function.

**Conclusion:**

Both lncRNA TINCR and the rs2288947 polymorphism contribute to NPDR development and show promise as biomarkers for early screening and risk evaluation.

## 1. Introduction

Diabetic retinopathy (DR), a major microvascular complication of diabetes, represents a leading cause of visual impairment and blindness in adults globally. Within the Chinese Han population, its prevalence and severity are notably high, which has been attributed to the interplay of genetic predisposition and environmental influences [1–3]. The key pathological mechanisms underlying DR involve hyperglycemia‐induced damage to retinal microvascular endothelial cells, dysregulated vascular growth, and subsequent fibrotic changes [4, 5]. Recent evidence highlights the important regulatory functions of long noncoding RNAs (lncRNAs) in modulating epigenetic, transcriptional, and posttranscriptional events. These molecules are increasingly recognized for their roles in endothelial cell function, inflammatory response, and angiogenesis. Thus, changes in lncRNA expressions may offer promise as early diagnostic markers and novel therapeutic targets [6–9]. Despite these advances, the involvement of specific lncRNAs, such as TINCR, in DR pathogenesis and their potential utility in diagnosis remain inadequately explored and merit further study.

Genetic susceptibility to DR involves variations such as expression quantitative trait loci (eQTLs) and other genetic polymorphisms that influence gene expression. For example, specific variants in the folliculin (FLCN) gene have been linked to increased risk of DR [10, 11]. Within the Chinese Han population, interactions between genetic and environmental factors are likely to accelerate DR progression. Nevertheless, research on certain genetic polymorphisms, such as rs2288947 in the lncRNA TINCR, remains limited. Notably, lncRNA TINCR has been implicated in various pathological processes; it attenuates cardiac hypertrophy via the miR‐211–3p/VEGFB pathway in heart disease and participates in epigenetic regulation in breast cancer, indicating its potential broad role in diabetic complications [12, 13]. Moreover, METTL14‐mediated m^6^A methylation has been shown to influence NLRP3‐dependent pyroptosis by modulating lncRNA TINRNA stability, a process already described in diabetic cardiomyopathy, suggesting a conceivable role for this mechanism in the vascular and neuronal injury observed in DR [14].

From a diagnostic standpoint, the early identification of DR depends critically on the availability of dependable molecular biomarkers. lncRNAs represent promising candidates in this regard, owing to their tissue‐specific expression patterns and measurable presence in serum. Previous studies have reported elevated levels of serum lncRNA OGRU in individuals with DR, with its expression corresponding to disease severity, thereby underscoring the value of quantitative lncRNA assays as an auxiliary diagnostic tool [15]. While the function of lncRNA TINCR in DR remains insufficiently explored, its documented upregulation in malignancies and modulatory impact on inflammatory pathways imply a potential role in the alteration of the retinal microenvironment [14, 15].

In summary, the current investigation examines the relationship between the rs2288947 polymorphism in lncRNA TINCR and genetic susceptibility to DR within a Chinese Han cohort. In addition, it assesses the potential of circulating lncRNA TINCR as a diagnostic indicator. The findings aim to bridge current gaps in understanding and offer novel perspectives for tailored preventive strategies and early management of DR.

## 2. Method

### 2.1. Study Participants

This study was conducted with the approval of the Ethics Committee of Dongying People’s Hospital, and written informed consent was acquired from each participant. 252 individuals with Type 2 diabetes mellitus (T2DM) and 255 patients diagnosed with nonproliferative DR (NPDR) were consecutively recruited from the Department of Endocrinology and Ophthalmology at our institution. All individuals with T2DM were diagnosed in accordance with the World Health Organization (WHO) criteria set forth in 1999. The diagnosis of NPDR was established according to the International Clinical DR Severity Scale. All enrolled patients underwent color fundus photography and/or fluorescein angiography. Two fundus disease specialists, who were masked to the clinical data, independently reviewed the images to determine the presence of NPDR. The diagnostic criteria required the identification of typical NPDR signs, including microaneurysms, intraretinal hemorrhages, hard exudates, and cotton‐wool spots, in the absence of PDR manifestations such as optic disc neovascularization (NVD), retinal neovascularization elsewhere (NVE), vitreous hemorrhage, or preretinal hemorrhage. In cases of disagreement between the two specialists, a consensus was reached through discussion or adjudication by a third senior expert. This study specifically focused on the presence or absence of NPDR and did not further stratify cases into mild, moderate, or severe categories.

The control group comprised T2DM patients with no evidence of retinal abnormalities, as confirmed through comprehensive ophthalmic assessment. Exclusion criteria included Type 1 diabetes, other types of retinopathy, significant hepatic or renal impairment, malignant tumors, current pregnancy, and acute infectious conditions.

### 2.2. Genotyping

Genomic DNA was isolated from peripheral blood samples employing QIAGEN extraction kits. SNP genotyping was performed using a predesigned TaqMan assay (Assay ID: C_7,738,215_1) on the QuantStudio 7 Flex Real‐Time PCR platform (Applied Biosystems, USA). In the present study, the overall average success rate for genotyping across all samples was determined to be 99.2%, with individual SNP loci achieving a rate exceeding 98%. To ensure data reliability, samples or loci exhibiting a call rate below 95% were deemed unreliable and consequently excluded from all subsequent analyses. For the purpose of error rate estimation, approximately 10% of the samples were randomly selected and subjected to a blinded duplicate genotyping procedure. A comparison of the resulting data revealed a concordance rate surpassing 99.5%, which translates to an experimental error rate of less than 0.5%. This high level of reproducibility robustly validates the reliability of the TaqMan genotyping assay employed.

### 2.3. Quantitative Real‐Time PCR (qRT‐PCR)

Fasting venous blood was collected from each participant in the morning using EDTA‐anticoagulated tubes. Plasma isolation was carried out by centrifuging samples at 3000 rpm for 10 min. Total RNA was isolated with TRIzol LS reagent (Invitrogen, USA), and cDNA was synthesized via reverse transcription employing the PrimeScript RT Reagent Kit (Takara, Japan). The extracted RNA was determined for its concentration and purity using NanoDrop 2000. Only those samples with an A260/280 ratio between 1.8 and 2.0 and a concentration of ≥ 10 ng/μL were included. qRT‐PCR was conducted with SYBR Premix Ex Taq II (Takara, Japan) on a LightCycler 480 II instrument (Roche, Switzerland). The geNorm and NormFinder algorithms were employed to assess the stability of candidate reference genes. GAPDH was identified as the most stably expressed gene, exhibiting the lowest geNorm M‐value (*M* = 0.35). Furthermore, the selection of GAPDH as the reference gene aligns with methodologies used in prior studies on serum lncRNA biomarkers for DR [9]. Therefore, GAPDH served as the internal control, and the relative expression of TINCR was determined using the 2^−ΔΔCt^ method. The forward primer for GAPDH is5′‐TGC​ACC​ACC​AAC​TGC​TTA​GC‐3′,and the reverse primer is5′‐GGC​ATG​GAC​TGT​GGT​CAT​GAG‐3′. TINCR forward primer 5′‐GAA​GCG​CTA​CCA​CAT​CAA​GG‐3′, reverse primer 5′‐CAC​CGT​CTG​GTG​GTC​GTC‐3′.

### 2.4. Data Analysis

Statistical analyses were conducted with SPSS 24.0 and GraphPad Prism 9.0. Continuous variables were summarized as mean ± standard deviation (Mean ± SD) and compared using Student’s *t*‐test or one‐way ANOVA, as appropriate. Categorical data were described as frequency counts and percentages (*n* [%]), and group differences were assessed with the chi‐square test. Genotype distribution conformity to Hardy–Weinberg equilibrium (HWE) was verified via the SHEsis online tool. Independent risk factors for DR were identified using multivariate logistic regression. All variables were simultaneously entered into the model using the enter method (forced entry method) to avoid bias caused by stepwise regression. The adjusted odds ratios (ORs) and their 95% confidence intervals (CIs) of each variable were calculated. To test for multicollinearity among the variables, the variance inflation factor (VIF) was calculated. A VIF value of less than 5 was considered as no significant multicollinearity. The diagnostic performance was evaluated by receiver operating characteristic (ROC) curve analysis. A two‐tailed *p* value below 0.05 was deemed statistically significant.

## 3. Results

### 3.1. Basic Demographic and Clinical Features of the Study Participants

As indicated in Table 1, this study enrolled 252 individuals with T2DM and 255 patients diagnosed with DR. The two groups were comparable in terms of age, gender, body mass index (BMI), systolic blood pressure (SBP), diastolic blood pressure (DBP), as well as triglyceride (TG), total cholesterol (TC), and low‐density lipoprotein cholesterol (LDL‐C) levels (all *p* > 0.05). However, compared to the T2DM group, subjects with DR exhibited a significantly longer duration of diabetes and higher glycated hemoglobin (HbA1c) values. In addition, the DR group showed lower high‐density lipoprotein cholesterol (HDL‐C), elevated serum creatinine, and a markedly lower estimated glomerular filtration rate (eGFR). These results suggest that patients with DR, relative to those with T2DM alone, tend to have a longer history of diabetes, worse glycemic management, reduced HDL‐C, and more advanced renal dysfunction.

**TABLE 1:** Comparison of baseline characteristics and clinical parameters between patients with T2DM and DR.

Items	T2DM (*n* = 252)	DR (*n* = 255)	*p* value
Age (years)	61.54 ± 9.90	60.60 ± 9.61	0.283
Gender (*n*/%)			0.758
Male	127/50.4	132/51.8	
Female	125/49.6	123/48.2	
BMI (kg/m^2^)	23.24 ± 2.00	22.94 ± 2.17	0.102
Duration of diabetes	9.26 ± 4.12	12.35 ± 4.16	< 0.001
HbA1c, % (mmol/mol)	8.03 ± 0.87	8.42 ± 2.13	0.007
SBP (mmHg)	131.82 ± 30.06	135.70 ± 22.69	0.102
DBP (mmHg)	77.01 ± 13.75	78.81 ± 11.97	0.116
TG (mmol/L)	2.38 ± 1.05	2.54 ± 1.28	0.139
TC (mmol/L)	4.90 ± 1.34	5.02 ± 1.26	0.279
HDL‐C (mmol/L)	1.20 ± 0.33	1.14 ± 0.31	0.034
LDL‐C (mmol/L)	2.75 ± 1.09	2.86 ± 1.05	0.226
Serum creatinine (mg/dL)	1.40 ± 0.74	1.54 ± 0.83	0.038
eGFR (ml/min/1.73 m^2^)	93.95 ± 21.19	88.17 ± 33.74	0.020

*Note:* T2DM, Type 2 diabetes mellitus; TG, triglyceride; HbA1c, hemoglobin A1c.

Abbreviations: BMI, body mass index; DBP, diastolic blood pressure; DR, diabetic retinopathy; eGFR, estimated glomerular filtration rate; HDL‐C, high‐density lipoprotein cholesterol; LDL‐C, low‐density lipoprotein cholesterol; SBP, systolic blood pressure; TC, total cholesterol.

### 3.2. Expression Profile and Diagnostic Efficacy of LncRNA TINCR in Patients With DR

This investigation measured the expression of lncRNA TINCR in peripheral blood samples obtained from individuals with T2DM and DR. As illustrated in Figure 1(a), expression levels of lncRNA TINCR were markedly elevated in the DR group relative to the T2DM group, implying a possible role in the development and advancement of DR. Moreover, multivariate logistic regression analysis, which accounted for clinical variables including disease duration, HbA1c, and HDL, identified lncRNA TINCR and rs2288947 as independent risk factors for DR (Figure 1(b) and Supporting Table 1). In addition, the VIFs for all the variables were all less than 5. The maximum value was 1.013. This indicated that there was no issue with multicollinearity in the model (Supporting Table 2). To assess its diagnostic potential, a ROC curve was constructed (Figure 1(c)). The area under the curve (AUC) achieved was 0.915 and 95% CI: 0.8902–0.9395, indicating high diagnostic accuracy. At the optimal threshold value of 1.264, lncRNA TINCR exhibited a sensitivity of 84.3% and a specificity of 87.3%. The robustness of the univariable lncRNA TINCR diagnostic model was validated internally using 10‐fold cross‐validation and 1000 bootstrap resamples. Model calibration was evaluated through calibration curves and the Hosmer–Lemeshow test, with results presented in Supporting Figure 1. The initial lncRNA‐based model yielded a diagnostic AUC of 0.915. Following validation, the mean AUC was 0.9161 (95% CI: 0.8507–0.9892) for the 10‐fold cross‐validation and 0.9149 (95% CI: 0.9149–0.9149) for the bootstrap method, indicating no significant overfitting (Supporting Figures 1A and 1B). The calibration curve demonstrated strong concordance between predicted and observed probabilities, and the Hosmer–Lemeshow test (*p* = 0.0521) confirmed adequate model calibration (Supporting Figure 1C).

FIGURE 1Expression, clinical significance, and diagnostic value of lncRNA TINCR in DR. (a) Relative expression levels of lncRNA TINCR in peripheral blood samples from patients with T2DM and patients with DR. (b) Forest plot of multivariate logistic regression analysis for factors associated with DR. (c) ROC curve analysis to evaluate the diagnostic performance of lncRNA TINCR for DR.

**Figure:**
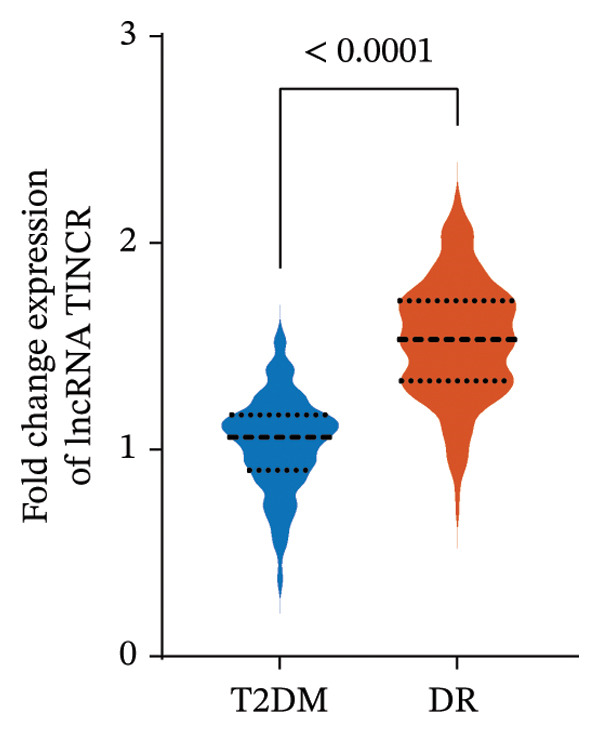
(a)

**Figure:**
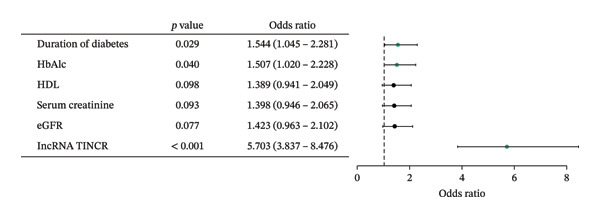
(b)

**Figure:**
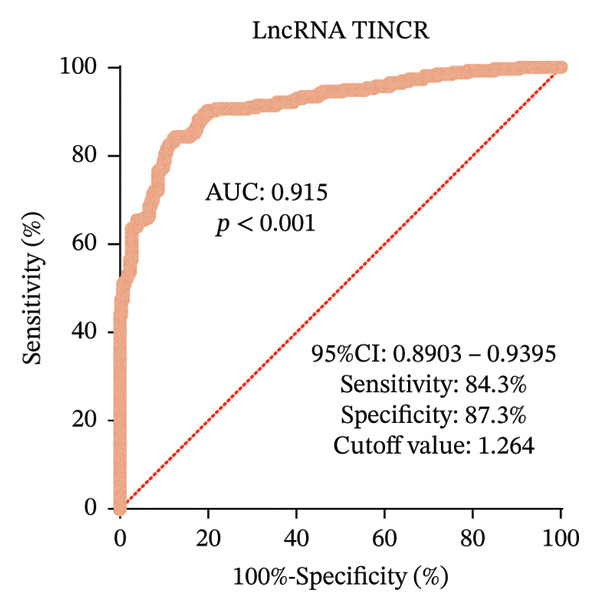
(c)

### 3.3. Association Between rs2288947 and Susceptibility to DR

Based on the association analysis of the rs2288947 polymorphism and the risk of DR, the statistical power was evaluated using the recessive model with the strongest effect and the most stringent statistical requirements (GG vs. AA + GA). The frequency of the GG genotype in the control group was 6.0% (15/252), and in the case group, it was 18.4% (47/255), with an OR of 3.57. With the current sample size (252 cases in the control group and 255 cases in the case group), at a bilateral *α* = 0.05 level, the power to detect this association was > 99.9% (PASS 15.0 software). Considering the multiple tests of the dominant, recessive, and co‐dominant genetic models, after Bonferroni correction, the significance level was *α*′ = 0.017. At this time, the power to detect the recessive model with an OR of 3.57 was still 97.8%, and the power of other models (allele OR = 1.83, dominant OR = 1.76) was all > 95%. This indicates that the sample size of this study is sufficient, and the found association results are robust and reliable.

This study examined the relationship between the genetic variant rs2288947 and the risk of DR by comparing its distribution in individuals with T2DM and those who had developed DR. A significant association was observed between this locus and susceptibility to DR (Table 2). The frequency of the *G* allele in the control group was 0.264, which was consistent with the frequency of the East Asian population in gnomAD (EAS: 0.277). HWE was tested in both the case group and the control group, with *p* values of 0.067 and 0.408, respectively. Both values were greater than 0.05, indicating no genotype bias. Under the co‐dominant genetic model, the GG genotype was more prevalent in the DR group compared to the T2DM group (18.4% vs. 6.0%), yielding an OR of 4.199 (95% CI: 2.222–7.934, *p* < 0.001), indicating that the GG genotype may contribute to increased DR risk. Analysis of allele frequencies showed a higher frequency of the *G* allele in the DR group relative to the T2DM group (39.6% vs. 26.4%), with an OR of 1.829 (95% CI: 1.402–2.387, *p* < 0.001). Additional analyses using dominant (GG + AG vs. AA) and recessive (GG vs. AA + AG) models further supported the role of the *G* allele in elevating DR risk, with ORs of 1.760 (95% CI: 1.237–2.505, *p* = 0.002) and 3.570 (95% CI: 1.939–6.573, *p* < 0.001), respectively. Moreover, genotype distributions in all groups conformed to HWE (*p* = 0.408), suggesting that the sampled population was genetically representative. In summary, the *G* allele, especially in the homozygous GG form at locus rs2288947, is significantly associated with an elevated risk of DR.

**TABLE 2:** Association analysis between the rs2288947 polymorphism and the risk of DR in T2DM.

Rs 2,288,947	T2DM (*n* = 252)	NPDR (*n* = 255)	*χ*2	OR (95% CI)	*p*
*Co-dominant model (n/%)*					
AA	134 (53.2)	100 (39.2)	—	1	—
GA	103 (40.8)	108 (42.4)	3.182	1.405 (0.967–2.042)	0.074
GG	15 (6.0)	47 (18.4)	21.444	4.199 (2.222–7.934)	< 0.001

*Alleles*					
A	371 (73.6)	308 (60.4)	—	1	
G	133 (26.4)	202 (39.6)	20.022	1.829 (1.402–2.387)	< 0.001

*Dominant model (n/%)*					
AA	134 (53.2)	100 (39.2)	—	1	—
GA + GG	118 (46.8)	155 (60.8)	9.937	1.760 (1.237–2.505)	0.002

*Recessive model (n/%)*					
AA + GA	237 (94.0)	208 (81.6)	—	1	—
GG	15 (6.0)	47 (18.4)	18.389	3.570 (1.939–6.573)	< 0.001
*P* ^HWE^	0.408				

*Note:* T2DM, Type 2 diabetes mellitus.

Abbreviations: HWE, Hardy–Weinberg equilibrium; NPDR, nonproliferative diabetic retinopathy.

### 3.4. Relationship Between LncRNA TINCR rs2288947 Polymorphism and Clinical Characteristics in Patients

As indicated in Table 3, subjects were categorized according to the lncRNA TINCR rs2288947 genotype into two groups, AA (*n* = 100) and GA + GG (*n* = 155). Comparative analysis of clinical parameters revealed no statistically significant differences in age, gender, BMI, SBP, DBP, TG, TC, or LDL‐C between the groups. However, individuals carrying the GA/GG genotypes exhibited a significantly longer duration of diabetes, higher HbA1c levels, reduced HDL‐C, elevated serum creatinine, and lower eGFR compared to those with the AA genotype. These results imply that the GA/GG genotype may be linked to an extended duration of diabetes, suboptimal glycemic control, altered lipid metabolism, and reduced renal function.

**TABLE 3:** Association between the lncRNA TINCR rs2288947 polymorphism and DR patients’ clinical characteristics.

Items	LncRNA TINCR rs2288947		*p* value
	AA(*n* = 100)	GA + GG (*n* = 155)	
Age (years)	60.66 ± 9.55	60.56 ± 9.67	0.940
Gender (*n*/%)			0.751
Male	53/53.0	79/51.0	
Female	47/47.0	76/49.0	
BMI (kg/m^2^)	22.88 ± 2.14	22.98 ± 2.19	0.719
Duration of diabetes	10.49 ± 3.63	13.56 ± 4.05	< 0.001
HbA1c,% (mmol/mol)	7.77 ± 2.10	8.84 ± 2.04	< 0.001
SBP (mmHg)	133.42 ± 18.39	137.17 ± 25.02	0.170
DBP (mmHg)	77.26 ± 12.61	79.81 ± 11.47	0.097
TG (mmol/L)	2.37 ± 1.06	2.63 ± 1.42	0.085
TC (mmol/L)	4.94 ± 1.27	5.07 ± 1.25	0.413
HDL‐C (mmol/L)	1.22 ± 0.27	1.08 ± 0.32	< 0.001
LDL‐C (mmol/L)	2.71 ± 0.92	2.96 ± 1.13	0.069
Serum creatinine (mg/dL)	1.33 ± 0.76	1.66 ± 0.87	0.002
eGFR (ml/min/1.73 m^2^)	93.79 ± 29.39	84.46 ± 35.89	0.024

*Note:* TG, triglyceride; HbA1c, hemoglobin A1c.

Abbreviations: BMI, body mass index; DBP, diastolic blood pressure; eGFR, estimated glomerular filtration rate; HDL‐C, high‐density lipoprotein cholesterol; LDL‐C, low‐density lipoprotein cholesterol; SBP, systolic blood pressure; TC, total cholesterol.

### 3.5. Association of the RS2288947 Genotype With LncRNA TINCR Expression Levels in T2DM and NPDR

qRT‐PCR results indicated that among patients with T2DM, those with the AA genotype at the rs2288947 locus showed reduced expression levels of lncRNA TINCR relative to carriers of the GA or GG genotypes (Figure 2(a)). This pattern was further corroborated in individuals diagnosed with DR, where the AA genotype group likewise exhibited markedly decreased lncRNA TINCR expression compared to the GA/GG group (Figure 2(b)). Collectively, these findings indicate that the rs2288947 genetic variation may contribute to the pathogenesis of T2DM and DR by modulating lncRNA TINCR expression. The AA genotype could potentially attenuate disease progression via inhibiting lncRNA TINCR, and future in silico functional studies are needed to verify these associations.

FIGURE 2Relative expression levels of lncRNA TINCR in patients with T2DM and DR stratified by rs2288947 genotype. (a) In patients with T2DM, the relative expression level of lncRNA TINCR in individuals carrying the AA genotype was reduced compared to that in patients with the GA or GG genotype. (b) In patients diagnosed with DR, the AA genotype group exhibited markedly lower relative expression of lncRNA TINCR than the GA/GG genotype group.

**Figure:**
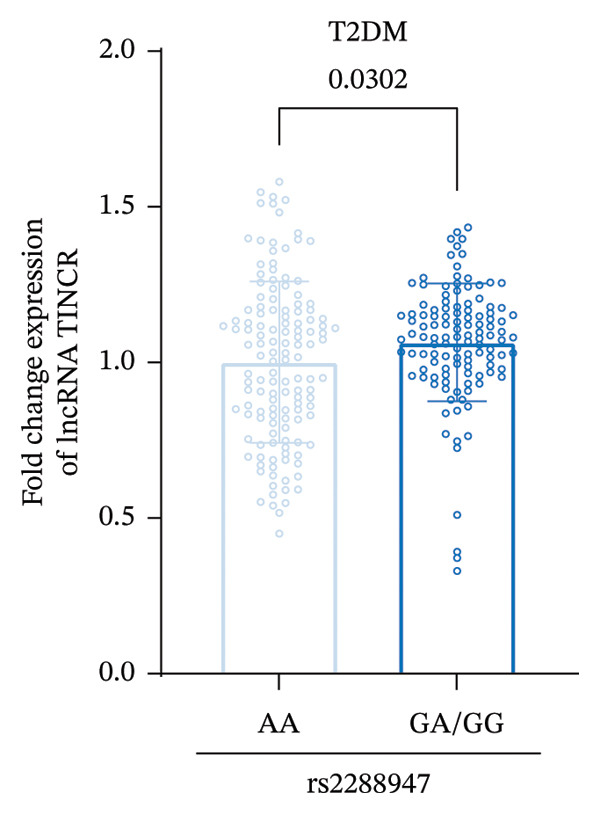
(a)

**Figure:**
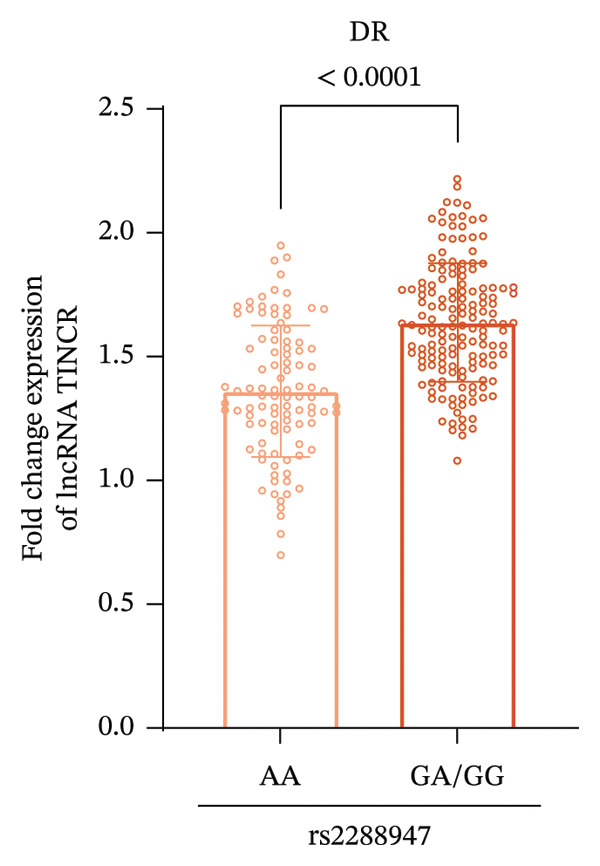
(b)

## 4. Discussion

DR, a prevalent and severe microvascular complication associated with T2DM, remains a major contributor to adult vision impairment worldwide [1, 16]. The development of DR involves multifactorial pathogenesis, encompassing genetic predisposition, metabolic dysregulation, and alterations in molecular signaling pathways. Recent evidence highlights the significance of lncRNAs in modulating the initiation and advancement of DR [6, 9, 17]. This investigation examines the lncRNA TINCR and its genetic variant rs2288947, offering a comprehensive analysis of their expression patterns, association with genetic susceptibility, and clinical relevance among T2DM patients with DR. The results contribute to novel insights into the molecular basis of DR and suggest potential diagnostic biomarkers.

In this study, the clinical profiles of patients with T2DM and those with DR were compared based on a set of basic parameters. The analysis revealed no statistically significant differences in age, sex distribution, blood pressure, or the majority of lipid profiles between the two groups. However, individuals in the DR group were found to have a longer history of diabetes, elevated HbA1c, reduced HDL‐C, and impaired renal function, as evidenced by increased serum creatinine levels and a lower eGFR. These observations align with earlier research [18–21], reinforcing that prolonged hyperglycemia, dyslipidemia, and renal dysfunction are critical factors contributing to the onset and advancement of DR.

This investigation provides the first molecular‐level evidence that lncRNA TINCR is markedly upregulated in the peripheral blood of individuals with DR. Multivariate logistic regression analysis confirmed lncRNA TINCR as an independent risk factor for the onset of DR. Furthermore, ROC curve assessment revealed that lncRNA TINCR possesses strong diagnostic accuracy for DR, with sensitivity and specificity each surpassing 84%. These results indicate that measuring its expression in peripheral blood could offer a noninvasive approach to assist in the screening and diagnosis of DR. These observations align with previous reports highlighting the diagnostic potential of various lncRNAs in DR [4, 15, 22]. Although lncRNA TINCR has previously been associated with tumors and metabolic disorders where it modulates processes such as cell proliferation, apoptosis, and inflammation [12, 23–25], this study expands its role to include diabetic microvascular complications. Its increased expression may indicate changes in epigenetic regulation resulting from prolonged hyperglycemia, potentially affecting downstream genes via a competitive endogenous RNA (ceRNA) mechanism. This could promote pathogenic events such as vascular endothelial dysfunction and breakdown of the blood–retinal barrier. In support of this, other studies have indicated that elevated lncRNA expression correlates with diabetes‐related oxidative stress, whereas suppression of these molecules can confer retinal protection and decelerate the progression of DR [4].

At the genetic level, this investigation centered on the polymorphism located at the rs2288947 site in the lncRNA TINCR gene region, revealing a notable correlation with susceptibility to DR. Across co‐dominant, dominant, and recessive genetic models, the *G* allele, especially the GG genotype was consistently linked to an elevated risk of DR. Additional allelic frequency analysis further confirmed that the *G* allele acts as a genetic risk factor for DR. It is also important to note that the genotype distribution at this locus adhered to the HWE, reflecting a genetically stable sample population and thereby strengthening the validity of these outcomes. These results are consistent with the recognized role of lncRNAs in mechanisms involving genetic variation. For instance, polymorphisms within lncRNA regions can modify transcriptional efficiency or RNA binding affinity, ultimately affecting functional gene expression and contributing to disease pathogenesis [26]. Likewise, variations in lncRNAs have repeatedly been correlated with diabetic complications via the modulation of cellular processes, including proliferation, migration, and angiogenesis [4, 27]. When clinical parameters were stratified according to rs2288947 genotype, individuals with GA or GG genotypes were found to have a longer history of diabetes, elevated HbA1c levels, reduced HDL‐C, and impaired renal function markers, characteristics that align closely with the typical clinical presentation of DR. This suggests that the genetic variant is not only tied to molecular expression changes but may also facilitate DR development indirectly by affecting intermediate phenotypic traits such as glycemic stability, lipid metabolism, and renal performance. Further functional association studies indicated a strong relationship between rs2288947 genotypes and the expression levels of lncRNA TINCR. Specifically, in both T2DM and DR patients, those with the AA genotype showed markedly reduced lncRNA TINCR expression relative to carriers of the GA or GG genotypes. This implies that the rs2288947 locus may be situated within a regulatory segment that affects lncRNA TINCR expression through various potential mechanisms, such as alterations in transcription factor binding, chromatin architecture, or mRNA stability. Consequently, the GA/AA genotype might lead to increased lncRNA TINCR expression, attenuating its protective regulatory role and thereby accelerating disease progression.

Although rs2288947 has been bioinformatically predicted as a potential functional variant (RegulomeDB score 1d, with its linked rs4510145 influencing multiple transcription factor binding sites), its functional annotations are predominantly derived from nonocular tissues and should be interpreted cautiously without experimental validation in retinal contexts. Furthermore, the present study enrolled only patients with NPDR and did not include those with proliferative DR (PDR) or stratify NPDR by severity, limiting the assessment of its relationship with disease progression. To address these gaps, future investigations integrating retinal‐specific functional assays and analyses across the full spectrum of DR severity are warranted to elucidate the role of TINCR rs2288947 in DR pathogenesis.

In summary, this research comprehensively illustrates that lncRNA TINCR is upregulated in DR and underscores its utility as both an independent risk indicator and an effective diagnostic marker. Notably, this study is the first to identify a significant association between the genetic variant rs2288947 and susceptibility to DR, as well as its role in modulating lncRNA TINCR expression. These results advance the current knowledge of DR’s molecular pathogenesis and offer a conceptual framework for early detection of at‐risk individuals and the identification of new treatment targets. The combination of genetic profiling and molecular expression analytics holds promise for building more precise predictive tools for DR, which could support early preventive strategies and tailored complication management.

## Funding

The authors declare that no funds, grants, or other support were received during the preparation of this manuscript.

## Conflicts of Interest

The authors declare no conflicts of interest.

## Supporting Information

Additional supporting information can be found online in the Supporting Information section.

## Supporting information


**Supporting Information** Supporting Figure S1 presents the comprehensive performance evaluation of the diagnostic prediction model constructed in this study, which consists of three subpanels. Panel A displays the ROC curve of the model, with a mean AUC of 0.916 (95% CI: 0.8507–0.9821) obtained via 10‐fold cross‐validation, demonstrating excellent discriminative ability of the model. Panel B shows the results of 1000‐iteration bootstrap resampling validation, with a mean AUC of 0.9149 and a SD of *p* < 0.0001, confirming the robustness and stability of the model. Panel C illustrates the calibration curve of the model divided into 10 risk groups, where the observed positive probabilities are closely aligned with the perfect calibration line, indicating high consistency between the model’s predicted probabilities and actual observed outcomes. Supporting Table 1 shows the results of multivariate logistic regression analysis for independent influencing factors of NPDR. It is indicated that diabetes duration (OR = 1.544, 95% CI: 1.045–2.281, *p* = 0.029), HbA1c (OR = 1.507, 95% CI: 1.020–2.228, *p* = 0.04), and lncRNA TINCR (OR = 5.703, 95% CI: 3.837–8.476, *p* < 0.001) are independent influencing factors, while HDL‐C, serum creatinine, and eGFR have no significant independent effects on NPDR (all *p* > 0.05). Supporting Table 2 displays the VIF of multiple linear regression analysis between clinical variables and lncRNA TINCR. All VIF values are between 1.005 and 1.013, suggesting no obvious multicollinearity among the included variables, and the regression model is stable and reliable.

## Data Availability

The data that support the findings of this study are available from the corresponding author upon reasonable request.
