# Supplementary material for: LncRNA TINCR rs2288947 Polymorphism as a Genetic Susceptibility Factor for Diabetic Retinopathy
Source: J Ophthalmol. 2026 May 14;2026:2631055. doi: 10.1155/joph/2631055 (PMC13174572; doi:10.1155/joph/2631055)
Supplement: Supplementary file 1 — Supporting Information Supporting Figure S1 presents the comprehensive performance evaluation of the diagnostic prediction model constructed in this study, which consists of three subpanels. Panel A displays the ROC curve of the model, with a mean AUC of 0.916 (95% CI: 0.8507–0.9821) obtained via 10‐fold cross‐validation, demonstrating excellent discriminative ability of the model. Panel B shows the results of 1000‐iteration bootstrap resampling validation, with a mean AUC of 0.9149 and a SD of p < 0.0001, confirming the robustness and stability of the model. Panel C illustrates the calibration curve of the model divided into 10 risk groups, where the observed positive probabilities are closely aligned with the perfect calibration line, indicating high consistency between the model’s predicted probabilities and actual observed outcomes. Supporting Table 1 shows the results of multivariate logistic regression analysis for independent influencing factors of NPDR. It is indicated that diabetes duration (OR = 1.544, 95% CI: 1.045–2.281, p = 0.029), HbA1c (OR = 1.507, 95% CI: 1.020–2.228, p = 0.04), and lncRNA TINCR (OR = 5.703, 95% CI: 3.837–8.476, p < 0.001) are independent influencing factors, while HDL‐C, serum creatinine, and eGFR have no significant independent effects on NPDR (all p > 0.05). Supporting Table 2 displays the VIF of multiple linear regression analysis between clinical variables and lncRNA TINCR. All VIF values are between 1.005 and 1.013, suggesting no obvious multicollinearity among the included variables, and the regression model is stable and reliable. [file JOPH-2026-2631055-s001.zip › Supplementary Figure 1.pdf]

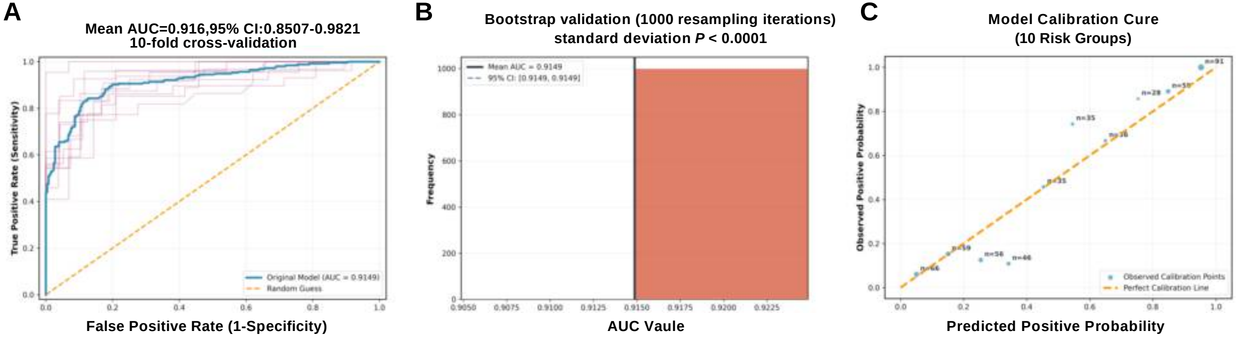
A
Mean AUC=0.916,95% CI:0.8507-0.9821
10-fold cross-validation
True Positive Rate (Sensitivity)
False Positive Rate (1-Specificity)
Original Model (AUC = 0.9149)
Random Guess
B
Bootstrap validation (1000 resampling iterations)
standard deviation P < 0.0001
Mean AUC = 0.9149
95% CI: [0.9149, 0.9149]
Frequency
AUC Vaule
C
Model Calibration Cure
(10 Risk Groups)
Observed Positive Probability
Predicted Positive Probability
Observed Calibration Points
Perfect Calibration Line
n=91
n=59
n=28
n=35
n=36
n=31
n=59
n=56
n=46
n=66
